# Facile Fabrication of High-Performance Thermochromic VO_2_-Based Films on Si for Application in Phase-Change Devices

**DOI:** 10.1021/acs.chemmater.3c00613

**Published:** 2023-05-30

**Authors:** Antonio J. Santos, Nicolas Martin, Juan J. Jiménez, Rodrigo Alcántara, Samuel Margueron, Andrea Casas-Acuña, Rafael García, Francisco M. Morales

**Affiliations:** † IMEYMAT: Institute of Research on Electron Microscopy and Materials of the University of Cádiz, Puerto Real E-11510, Spain; ‡ Department of Materials Science and Metallurgic Engineering, and Inorganic Chemistry, Faculty of Sciences, University of Cádiz, Puerto Real E-11510, Spain; § Université de Franche-Comté, CNRS, Institut FEMTO-ST, Besançon F-25000, France; ∥ Department of Physical Chemistry, Faculty of Sciences, University of Cádiz, Puerto Real E-11510, Spain

## Abstract

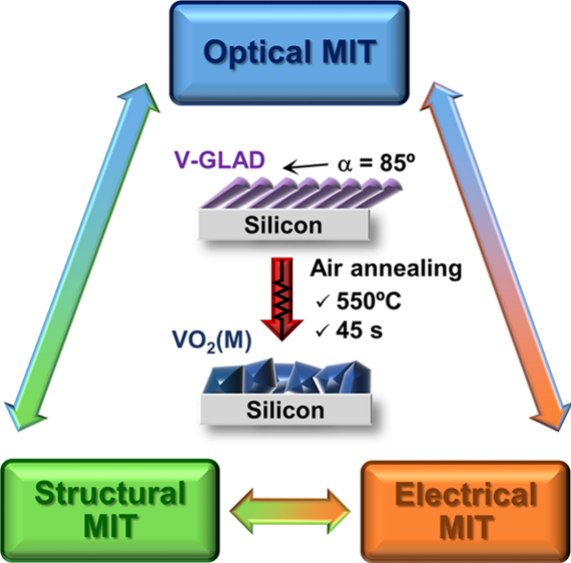

This work reports on an alternative and advantageous procedure to attain VO_2_-based thermochromic coatings on silicon substrates. It involves the sputtering of vanadium thin films at glancing angles and their subsequent fast annealing in an air atmosphere. By adjusting thickness and porosity of films as well as the thermal treatment parameters, high VO_2_(M) yields were achieved for 100, 200, and 300 nm thick layers treated at 475 and 550 °C for reaction times below 120 s. Comprehensive structural and compositional characterization by Raman spectroscopy, X-ray diffraction, and scanning-transmission electron microscopies combined with analytical techniques such as electron energy-loss spectroscopy bring to the fore the successful synthesis of VO_2_(M) + V_2_O_3_/V_6_O_13_/V_2_O_5_ mixtures. Likewise, a 200 nm thick coating consisting exclusively of VO_2_(M) is also achieved. Conversely, the functional characterization of these samples is addressed by variable temperature spectral reflectance and resistivity measurements. The best results are obtained for the VO_2_/Si sample with changes in reflectance of 30–65% in the near-infrared at temperatures between 25 and 110 °C. Similarly, it is also proven that the achieved mixtures of vanadium oxides can be advantageous for certain optical applications in specific infrared windows. Finally, the features of the different structural, optical, and electrical hysteresis loops associated with the metal–insulator transition of the VO_2_/Si sample are disclosed and compared. These remarkable thermochromic performances hereby accomplished highlight the suitability of these VO_2_-based coatings for applications in a wide range of optical, optoelectronic, and/or electronic smart devices.

## 1 Introduction

Since its discovery in 1959, vanadium dioxide (VO_2_) has been one of the most extensively studied functional materials mainly because it undergoes a reversible metal-to-insulator transition (MIT) at temperatures close to 68 °C.^1^ Driven by structural changes from insulating monoclinic (M) at low temperature to metallic rutile (R) at temperatures above the transition temperature (*T*_c_), this phenomenon involves great optical and electronic changes. This makes this material especially advantageous for thermochromic smart window applications.^2−7^ These appealing properties of VO_2_ have also been exploited for a wide range of photonic, optoelectronic, and electronic phase-change devices on silicon platforms, such as optical modulators and limiters,^8−13^ infrared (IR) photodetectors,^14,15^ or optical^16,17^ and electrical^18−21^ switches. Likewise, the VO_2_/Si system is also considered a potential candidate for its application as a passive intelligent radiator for spacecraft thermal control.^22^ However, the cost-effective attainment of VO_2_ films remains a challenge due, among other reasons, to its very narrow experimental synthesis window resulting from the complexity of the vanadium-oxygen system (e.g., existence of numerous and more thermodynamically stable oxides, different polymorphs for the same vanadium oxide, etc.), which generally translates into the formation of vanadium oxide mixtures.^23−26^

On the other hand, the characteristics of such an MIT transition (critical temperature, order of magnitude, hysteresis width) also depend on other factors such as film thickness,^27,28^ employed substrate,^29,30^ grain size distribution and boundaries,^31,32^ or crystallinity level.^33,34^ Therefore, the choice of one or another synthesis strategy becomes a decisive issue. Several one-step deposition techniques have been implemented to achieve VO_2_ nanostructures.^28,35−37^ Note that these procedures have obvious difficulties associated with finely controlling fluxes of O_2_, N_2_, and Ar, as well as with maintaining the required high temperatures (>450 °C) for long times. A widespread practice today to obtain high-quality VO_2_ films involves postdeposition annealing steps in an air atmosphere. The most commonly used precursors for this two-step approach are metallic vanadium^38−40^ and vanadium nitride.^41,42^

Within this framework, our previous study introduced an original two-step approach to synthesize VO_2_(M) on silicon substrates by means of a thermal oxidation process of porous vanadium films in an air atmosphere.^43^ Thanks to the fabrication of vanadium nanostructures with a high surface-to-volume area using the GLancing Angle Deposition (GLAD) technique combined with the subsequent implementation of fast and finely controlled thermal treatments, the selective synthesis of vanadium dioxide thicknesses (between 100 and 400 nm) with controlled grain sizes was accomplished. Nevertheless, the total layer thicknesses addressed in that work (∼600 nm) did not allow the fabrication of pure VO_2_ coatings, since, before all the remaining vanadium was completely oxidized, the dioxide formed was almost instantly transformed into other more oxygen-enriched oxides, such as V_2_O_5_. This resulted in 70% maximum VO_2_ yields of the total coating thickness. In addition, this study also lacked comprehensive structural/functional characterizations at variable temperatures, which are necessary to explore the MIT features of the resulting coatings.

To fill this gap, this work reports on the rapid air oxidation of porous vanadium films sputtered on silicon substrates from a more global perspective, assessing not only the effect of the different parameters involved in the annealing processes but also the influence of the layer thickness. With the ultimate aim of attaining pure VO_2_/Si films, direct current (DC) magnetron-sputtered V-GLAD layers of 100, 200, and 300 nm nominal thicknesses were deposited with a deposition angle α = 85°, which were subsequently annealed at different temperatures (*T*_r_) and reaction times (*t*_r_) depending on the volume of material to be oxidized. Comprehensive microstructural and compositional analyses of these oxidized systems were conducted by combining scanning electron microscopy (SEM), Raman spectroscopy (RS), grazing incidence X-ray diffraction (GIXRD), and scanning-transmission electron microscopy (S)TEM techniques, including high-angle annular dark-field imaging (HAADF) and high-resolution (HRTEM) imaging, as well as electron energy-loss spectroscopy (EELS). Once disclosed the role of both reaction parameters and layer thickness on the composition, morphology, and structure of the synthesized films, their functional characterization was addressed by means of variable temperature visible–near infrared (vis–NIR) reflectance and resistivity measurements within the range 25–110 °C, placing special emphasis on the effect of the different vanadium oxide mixtures on the resulting optical/electrical responses along the metal-to-insulator transition. Additionally, an exhaustive study on the structural (RS at variable temperature), optical, and electrical features of the MIT hysteresis of pure VO_2_/Si samples was also performed.

## 2 Materials and Methods

### 2.1 Deposition Process

Films were deposited at room temperature by DC magnetron sputtering from a vanadium metallic target (51 mm diameter and 99.9 atomic % purity) in a homemade deposition chamber. It was evacuated down to 10^–5^ Pa before each run by means of a turbomolecular pump backed by a primary pump. The target was sputtered with a constant current density of *J* = 100 A m^–2^, leading to a constant target potential of 312 V. Single-crystalline (100) *n*-type (P doped) silicon substrates were placed at a distance of 65 mm from the target center. On the basis of our previous studies,^43^ porous V films with large surface-to-volume ratios and enhanced reactivity with oxygen were deposited by the GLAD technique. The deposition angle α (the average angle of incoming particle flux) relative to the substrate normal was set at α = 85° (the maximum inclination allowed for efficient GLAD deposition, so that the greater the deposition angle, the higher the overall porosity of the film and, therefore, its specific surface area^44^) with no rotation of the substrate (i.e., ϕ = 0 rev h^–1^). Argon was injected at a mass flow rate of 2.40 sccm, and the pumping speed was maintained at *S* = 13.5 L s^–1^, leading to a sputtering pressure of 0.3 Pa. Different vanadium nominal thicknesses (100, 200, and 300 nm) were achieved by adjusting the deposition time according to an average deposition rate of 240 nm h^–1^, which was previously determined for α = 85°. The real thickness of vanadium films was measured in a Bruker DEKTAK XT 2D contact profilometer.

### 2.2 Thermal Treatments

After deposition, vanadium samples were thermally treated in a homemade reaction system. It consists in an Al_2_O_3_ tube on a SiC resistors furnace being able to reach temperatures up to 1500 °C, with an attached concentric steel tube and high-temperature steel-covered K-type thermocouple inside. This thermometer bar acts as an axle for a system of horizontal translation. At the end of the metallic tube nearby the furnace, the thermocouple crosses and fixes to a cylinder placed inside this tube, mechanized with a hitch to hang a combustion boat. Thus, the thermometer tip is always placed some millimeters over the center of this boat, which is an alumina crucible, allowing the temperature in the reaction zone to be life-tracked. The other end side also crosses and is fixed to another piece that is part of a handlebar used to slide the specimen holders inside and outside. In this way, by fixing a temperature in the center of the furnace, one is able to control the temperature increase (heating rate) by moving the boat more and more inside the furnace (for a more detailed overview of the reaction system, refer to previous studies^43,45,46^). Consequently, translation routines were prepared for reaching an average heating rate of 42 °C s^–1^, as well as for adjusting longer or shorter reaction times at a desired temperature. Lastly, all samples were cooled down in air.

### 2.3 Structural, Compositional, and Functional Characterizations

Topographic scanning electron microscopy (SEM) images were acquired using an FEI Nova NanoSEM microscope operating at 5 kV to examine the surface morphology of the films before and after each thermal treatment. Room-temperature Raman spectra were recorded on two different systems (HORIBA Scientific LabRAM HR Evolution and Jobin Yvon U1000) using 473 and 532 nm laser excitation sources with spectral resolutions of approximately 2 cm^–1^ in the range of 350–900 cm^–1^ (acquisition times of 20 s with an accumulation of 10 spectra) and 100–900 cm^–1^ (acquisition times of 8 s with an accumulation of 2 spectra), respectively. The power of the laser was controlled to avoid the degradation of the sample. Variable-temperature Raman measurements were conducted on a confocal Raman microspectrometer (Monovista, S-&-I GmbH) equipped with a THMS600 Linkam heating/cooling stage, setting the irradiated laser at 532 nm. The acquisition time was 60 s with an accumulation of two spectra (an average spectral resolution of 0.4 cm^–1^ in the range of 80–850 cm^–1^). GIXRD scans were performed on a Malvern Panalytical Aeris diffractometer (Cu radiation) working at 30 kV (10 mA) and setting a grazing incidence angle of 0.8°. High-resolution transmission electron microscopy (HRTEM) and high-angle annular dark-field imaging (HAADF) studies were carried out in a Thermo Scientific TALOS F200X G2 analytical microscope working at an accelerating voltage of 200 kV. A Gatan Imaging Filter (GIF) Continuum system fitted in the Talos microscope was used for spatially resolved electron energy-loss spectroscopy (EELS) analysis in scanning (STEM) mode. STEM-EELS 2D spectrum image (SI) data were acquired using a 2.5 mm diameter aperture and 0.05 eV/channel energy dispersion. The convergence and collection semiangles were set to 10.5 and 20.0 mrad, respectively, and the probe current was 150 nA. In this configuration, the energy resolution was 0.75 eV. To allow accurate chemical shift measurements, the Dual EELS mode was used to record nearly simultaneously both low-loss signal and the V-L_2,3_ and O-K high-loss edges, at each pixel position. Dwell times of about 0.3 s per pixel were set to optimize the signal-to-noise ratio. Electron-transparent cross-sectional samples were prepared for TEM observations in a Thermo Scientific Scios 2 DualBeam focused ion beam (FIB) system. The thermochromic optical behavior of the prepared VO_2_ films was determined via reflectance spectroscopy using a PerkinElmer Lambda 900 UV/vis/NIR Spectrometer equipped with a THMS600 Linkam stage for temperature control. Thus, vis–NIR reflectance spectra were recorded at selected temperatures in the wavelength range of 400–3300 nm, using silver as the mirror reference. DC electrical resistivity vs temperature measurements of the oxidized films were performed in a custom-made chamber. It is covered to have a dark environment, using the four-probe van der Pauw geometry in the temperature range of 25–100 °C with a ramp of 1 °C min^–1^ and then back to 25 °C with the same negative ramp. Humidity and cleanness were considered as constant. The error associated to all resistivity measurements was always below 1%, and the quality of the contacts was checked prior to every run (*I*/*V* correlation close to 1) to ensure that ohmic contacts were attained (use of gold-coated tips).

## 3 Results and Discussion

### 3.1 Morphology, Structure, and Composition of the Fabricated Films

In a first step, pure vanadium GLAD films of 100, 200, and 300 nm nominal thicknesses were sputtered on silicon substrates at α = 85°. This deposition configuration and layer thicknesses were selected to promote the subsequent rapid and selective oxidation of the entire coating in an air atmosphere, while maximizing VO_2_ yields. The real thicknesses of as-deposited V-GLAD films were evidenced by contact profilometry measurements performed in three different regions within the same sample. The results obtained are shown in Table 1. As can be seen, the real thicknesses move further and further away from the targeted ones as the total thickness of the film increases. This is attributed to the fact that, as layer thickness increases in GLAD systems, the deposition process becomes more dominated by events related to the shadowing effect so characteristic of this technique, so that only the larger columns progress to the detriment of the smaller ones.^47,48^ This phenomenon also leads to an increase in the overall porosity of the coating. As a result, the surface area in contact with the sputtered particle pathway becomes increasingly limited after exceeding a threshold thickness (the deposition process promoted exclusively by the shadowing effects), which can result in a decrease in the overall deposition rate.

**Table 1:** Nominal (τ_N_) and Real (τ_R_) V-GLAD Thicknesses for As-Deposited Samples Together with Their Annealing Conditions^a^

sample	τ_N_ (nm)	τ_R_ (nm)	*T*_r_ (°C)	*t*_r_ (s)
T100_475_60	100	97 ± 12	475	60
T100_550_1			550	1
T100_550_10				10
T100_550_30				30
T200_475_90	200	160 ± 16	475	90
T200_550_25			550	25
T200_550_45				45
T300_475_120	300	241 ± 21	475	120
T300_550_25			550	25
T300_550_45				45

a τ_R_ values were determined by contact profilometry. *T*_r_ is the reaction temperature, and *t*_r_ is the reaction time.

These fabricated systems were then subjected to different rapid thermal treatments. For this purpose, all samples were annealed at the same constant heating rate of 42 °C s^–1^ until reaching two reaction temperatures (*T*_r_) close to the limits of the operating window for the synthesis of VO_2_ in air: 475 and 550 °C. Such plateau temperatures are maintained for different reaction times (*t*_r_) to optimize VO_2_ yields as well as to achieve full oxidation of the different layer thicknesses addressed (the greater the V-GLAD volumes, the longer the oxidation times). Thereupon, samples are instantaneously cooled in air (for a detailed outline of the fast thermal treatment procedure, refer to our previous works^43,45,46^). All annealed samples are listed in Table 1 together with their treatment conditions and nomenclature.

**Figure 1:**
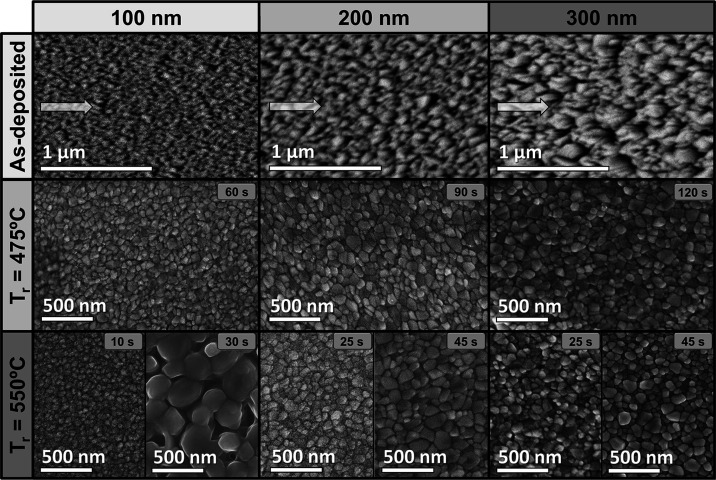
Topographic SEM micrographs of the deposited films before and after annealing. Arrows indicate the direction of the particle flux during each GLAD deposition.

Figure 1 shows the characteristic SEM topography of the fabricated coatings before and after heat treatment. Consistent with the above assumptions, the surface microstructure of as-deposited samples reveals a higher development of the overall porosity as the GLAD layer thickness increases, resulting in greater column diameters and surface roughness. On the other hand, oxidized samples generally exhibit a mosaic-like granular structure, which is characteristic of vanadium dioxide on silicon substrates.^43^ The formation of different grain sizes is also observed depending on the applied thermal treatment (Table 2). For the observation of the SEM topography of sample T100_550_1 and its grain size distribution, refer to the Supporting Information, Section I. As a general rule, it can be noted that longer reaction times lead to a progressive increase in grain size until reaching a maximum of 130–160 nm. The only sample that overcomes this barrier is T100_550_30, exhibiting about five times larger grain sizes when increasing the reaction time from 10 (51 ± 9 nm) to 30 s (230 ± 72 nm). Likewise, the morphology of these grains is also different in appearance from the other oxidized samples. This could be related to the formation of other vanadium oxides different from VO_2_ (probably with an O/V ratio higher than 2).

**Table 2:** Summary of the Average Grain Sizes for Thermally Treated Samples^a^

sample	grain size (nm)
T100_475_60	69 ± 21
T100_550_10	51 ± 9
T100_550_30	230 ± 72
T200_475_90	112 ± 46
T200_550_25	82 ± 22
T200_550_45	132 ± 31
T300_475_120	120 ± 35
T300_550_25	88 ± 15
T300_550_45	100 ± 28

a Note that these values were extracted from the analysis of different topographic SEM images.

On the other hand, it is also evidenced that, for the same *t*_r_ value, higher reaction temperatures result in larger grain sizes. In this regard, it must be noted that thermal treatments at 475 °C require more than twice the reaction time to achieve grain sizes similar to those obtained at 550 °C for the same sample thickness, the latter being another of the parameters that also plays an important role on this issue. Apart from the aforementioned exception, it is worth noting that the grain sizes for samples T100_550_10 and T100_475_60 do not exceed 90 nm. This could be due to the fact that 100 nm V-GLAD samples (note that from this point onward, only nominal layer thicknesses will be referenced to simplify and streamline the text) are the least porous, leading to smaller surface-to-volume areas. In other words, such samples are less susceptible to rapid and selective oxidation, hence the limitation of the grain size formed. In any case, it should be remembered that longer reaction times applied on 100 nm thick samples could lead to the undesired formation of other vanadium oxides different from the dioxide (sample T100_550_30).

**Figure 2:**
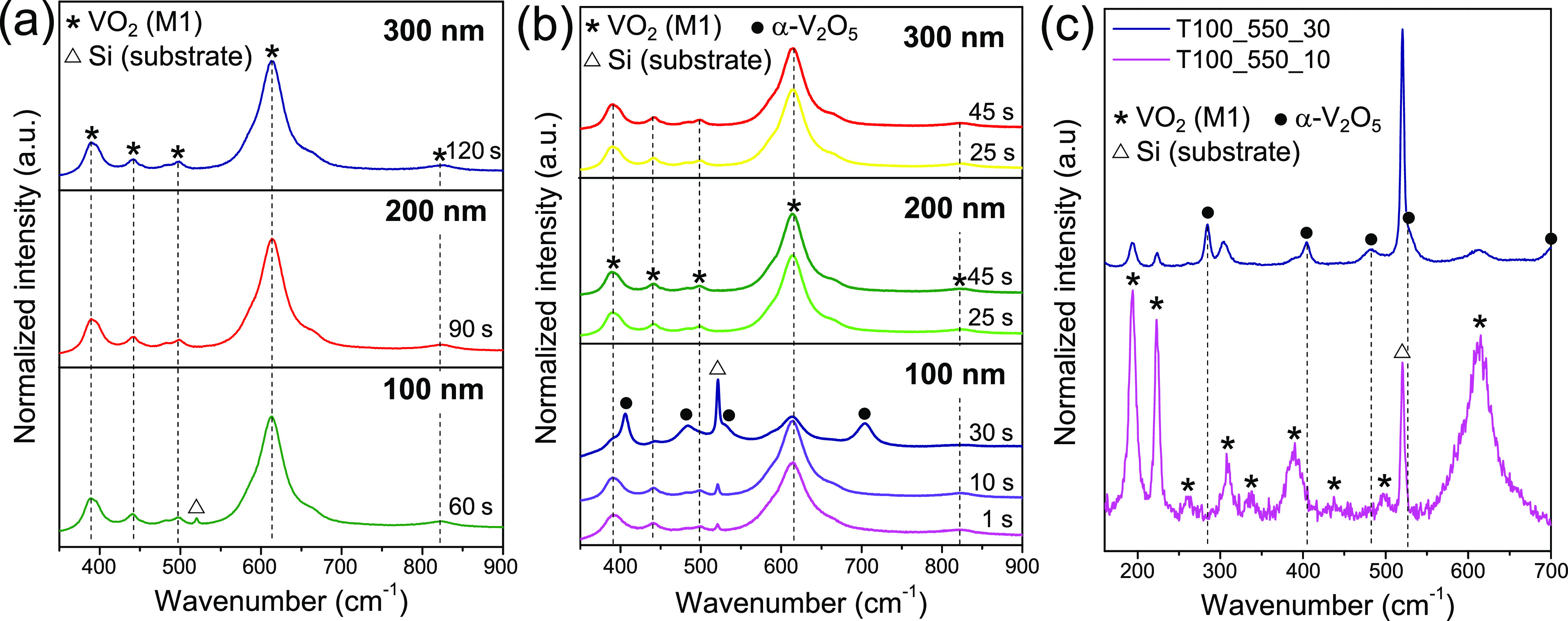
Room-temperature Raman spectra for all the samples annealed at (a) 475 °C and (b) 550 °C. (c) Raman spectra recorded for samples T100_550_10 (magenta) and T100_550_30 (blue) at 150–700 cm^–1^.

To corroborate the above, room-temperature Raman measurements were carried out for all samples oxidized at 475 and 550 °C. Results of these measurements are displayed in Figure 2a,b, respectively. They confirm the initial assessments so that, according to the Raman spectra reported in the literature for different vanadium oxides,^49^ there is no doubt that all samples contain VO_2_(M), with the exception of sample T100_550_30, which is composed of VO_2_(M) + α-V_2_O_5_ mixtures. For a better comparison between the two types of recorded signals, Figure 2c reveals the Raman spectra of samples T100_550_10 and T100_550_30 for a wider wavenumber range. Nonetheless, these outcomes should be interpreted with great care. This does not necessarily mean that neither the vast majority of samples consist exclusively of VO_2_(M) nor the entire film is completely oxidized. In this sense, it is worth noting that the Si (substrate) signal appears only for the 100 nm thick samples, being substantially broadened for VO_2_(M) + V_2_O_5_ mixtures (the latter having a greater transparency at visible wavelengths^50,51^), so it could be the case that the laser is not penetrating through the entire thickness of the film. In addition, there are oxides such as VO, V_2_O_3_, V_6_O_13_, or even metallic vanadium itself, which either do not give a Raman signal or it is very weak compared to that of the dioxide.^49^ Therefore, further investigations would be needed to better understand the structure and composition of these films.

**Figure 3:**
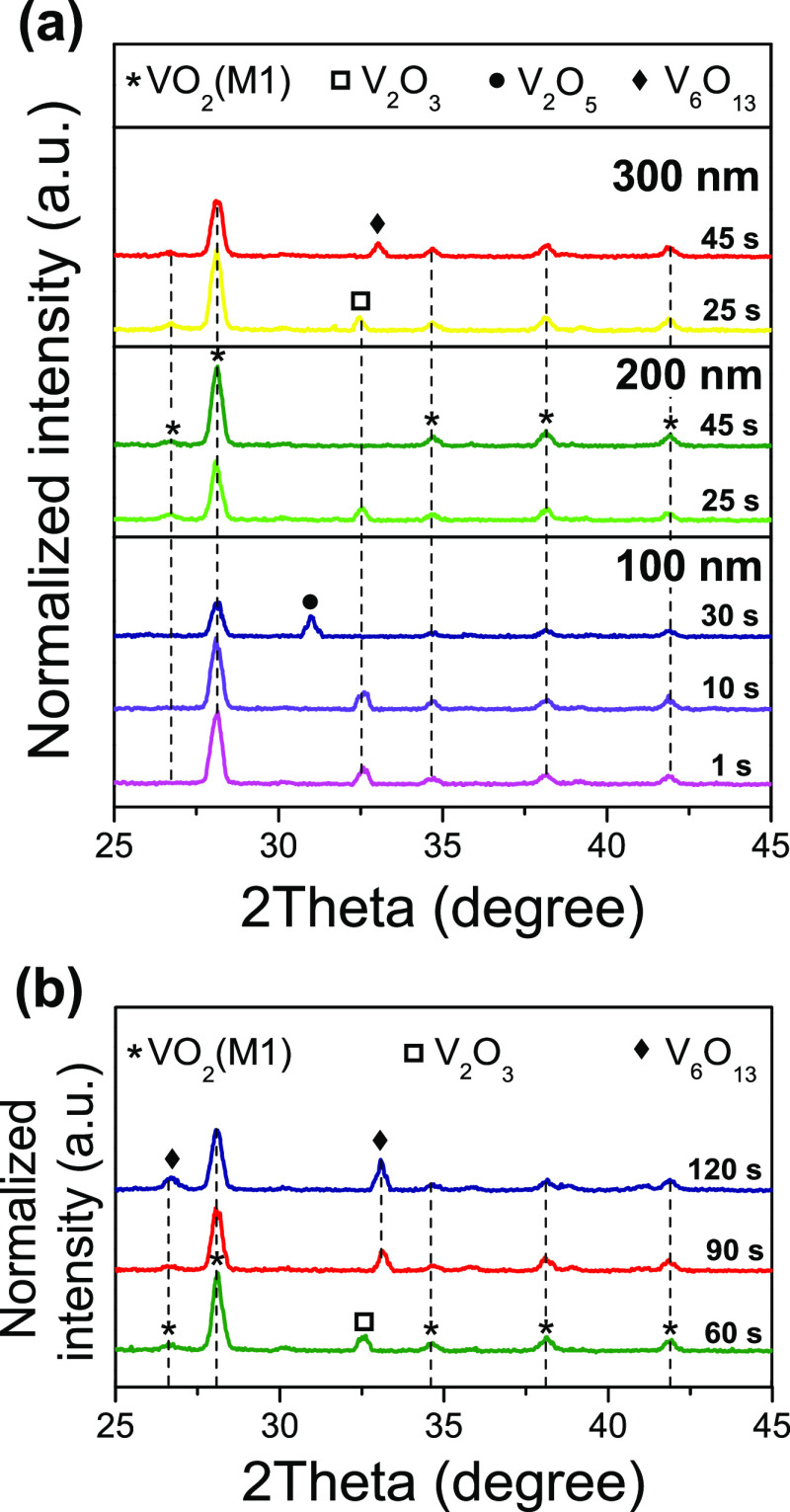
GIXRD diffractograms for V-GLAD samples annealed at (a) 550 °C and (b) 475 °C with reaction times (*t*_r_) ranging from 1 to 120 s. The thicknesses of the samples shown in panel b are 100 nm (green), 200 nm (red), and 300 nm (blue).

Figure 3 shows GIXRD diffractograms recorded for samples oxidized at 475 and 550 °C. These studies allowed to identify the presence of other oxides such as V_2_O_3_ (JCPDS Card No. 00-085-1411) and V_6_O_13_ (JCPDS Card No. 00-025-1251) forming mixtures with VO_2_(M) (JCPDS Card No. 03-065-2358), the latter being always present in all oxidized samples and thus in fine agreement with what was previously evidenced by RS. In this vein, VO_2_ crystallite sizes (from the full width at half maximum, FWHM, of peak at about 28° using the Scherrer equation) were determined to be 22–23 nm. It was also confirmed that sample T100_550_30 consists of VO_2_(M) + V_2_O_5_ (JCPDS Card No. 00-041-1426). Likewise, these analyses prove that metallic vanadium becomes fully oxidized even for instantaneous thermal treatments (sample T100_550_1), although giving rise to significant amounts of V_2_O_3_ (a vanadium oxide with an O/V ratio lower than that of VO_2_). Furthermore, it should be highlighted that sample T200_550_45 is the only one formed exclusively by VO_2_(M). All the others are formed by VO_2_ + V_2_O_3_ mixtures, which are identified in samples subjected to the lowest *t*_r_ for a given reaction temperature; or VO_2_(M) + V_6_O_13_ mixtures, for increasing layer thicknesses and/or reaction times. Special attention should be paid to sample T100_550_30, whose combination of *T*_r_, *t*_r_, and thickness seems to be the only one that overcomes the energy threshold for the activation of reactions leading to the synthesis of V_2_O_5_. In any case, it should be emphasized that the V-GLAD layer thicknesses as well as the reaction window selected here are suitable for synthesizing VO_2_ in air.

**Figure 4:**
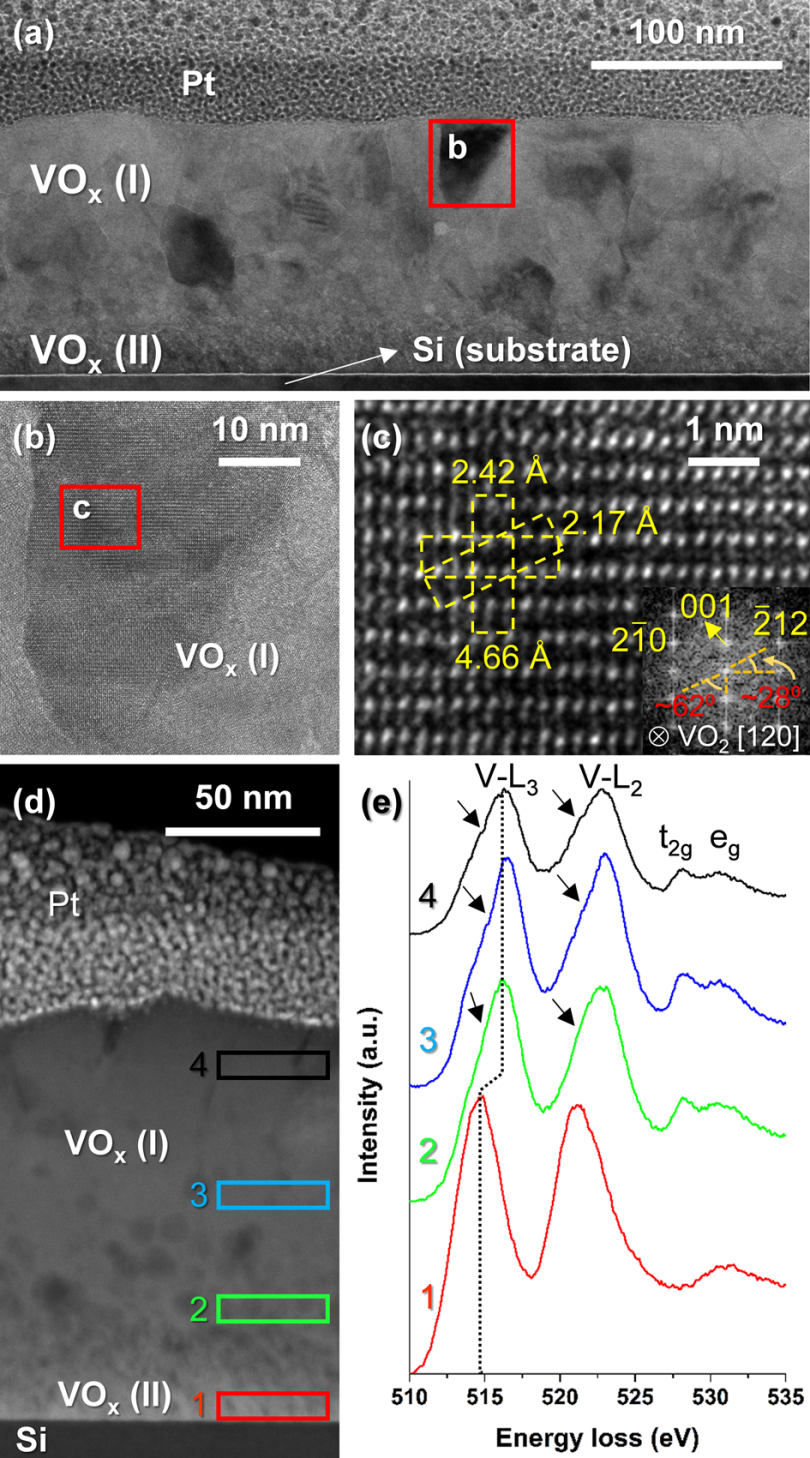
Analytical (S)TEM studies performed on sample T100_550_10. (a) Bright-field (BF) TEM overview of the sample taken with the sample oriented along one of the Si <110> zone axes. (b) High-resolution TEM micrograph depicting a grain belonging to the upper part of the film. (c) HRTEM micrograph of the narrow region highlighted in panel b together with its associated FFT spectrum. (d) High-angle annular dark-field (HAADF) overview of the sample in a new region different from that shown in panel a. (e) Integrated EELS spectra corresponding to the areas marked in panel d.

**Figure 5:**
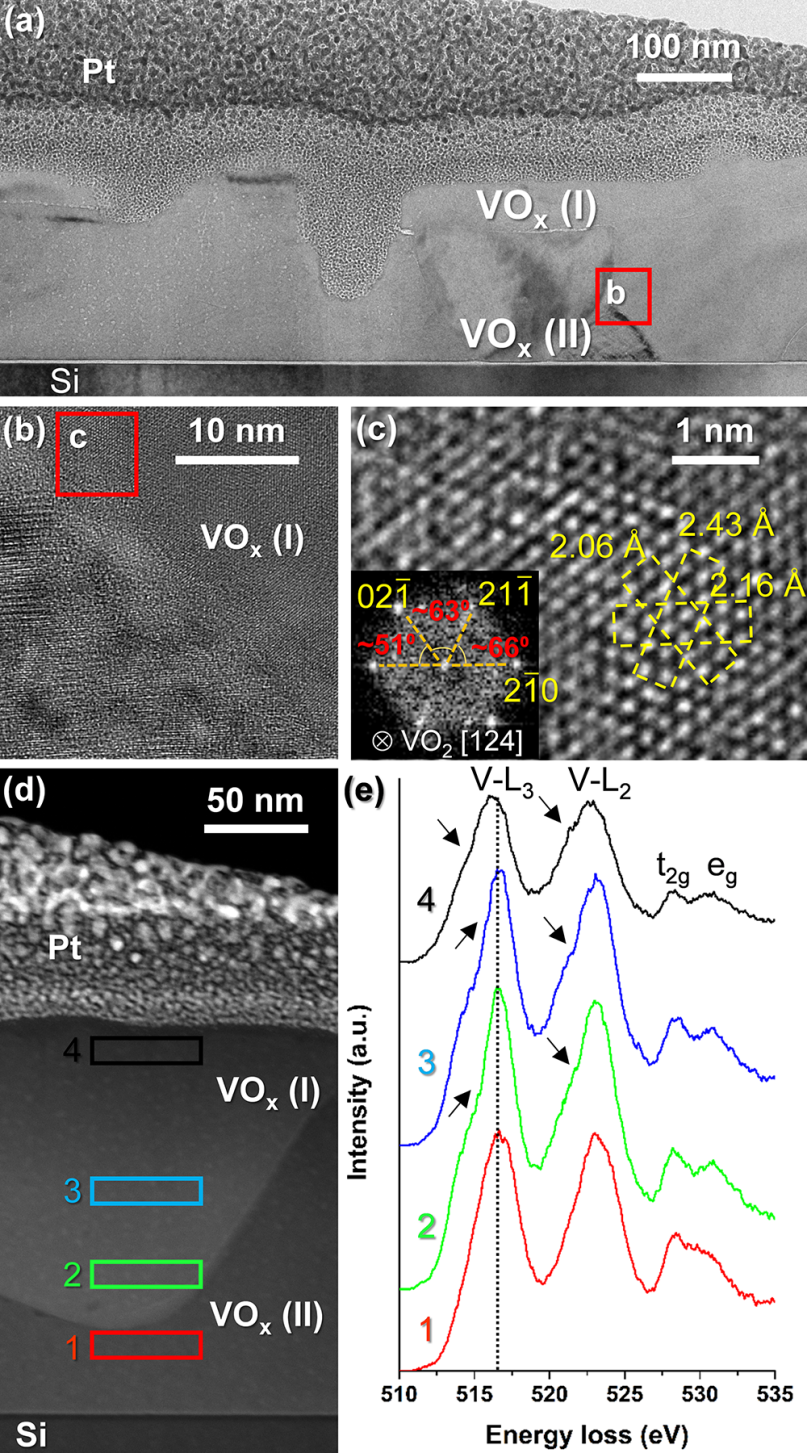
Analytical (S)TEM studies performed on sample T100_550_30. (a) Bright-field (BF) TEM overview of the sample along one of the Si <110> zone axes. (b) High-resolution TEM detail of the grain boundary zone. (c) HRTEM micrograph of the narrow region highlighted in panel b together with its associated FFT spectrum. (d) High-angle annular dark-field (HAADF) overview of the sample in a new region different from that shown in panel a. (e) Integrated EELS spectra corresponding to the areas marked in panel d.

With the aim of obtaining additional insights into the nanostructure and composition of these synthesized coatings as well as to better understand how oxidation processes take place, analytical (S)TEM studies were performed on samples T100_550_10 and T100_550_30. The results of these explorations are displayed in Figure 4 and Figure 5, respectively. Preliminarily, it can be noticed a considerable increase in the overall thickness of both coatings after thermal treatment (∼135 nm for T100_550_10 and ∼190 nm for T100_550_30 according to Figure 4a and Figure 5a, respectively). This is in line with our previous studies,^43^ which disclosed thicker samples as the coating became more oxygen enriched. In turn, this would also explain why sample T100_550_30, consisting of VO_*x*_ mixtures of higher valence states (V^4+^ and V^5+^), is thicker. Apart from that, Figure 4a allows to distinguish two clearly differentiated regions within the T100_550_10 sample. A first region with a relatively dense main layer, denoted as VO_*x*_ (I), formed by grains of size similar to those observed by SEM. A second one with a layer underneath of considerably lower thickness still preserves, albeit slightly, the tilted column geometry so characteristic of GLAD deposition (VO_*x*_ (II)). The latter suggests that this second region could be less oxidized, so that oxidation would occur from the film surface toward the interface with the substrate. Figure 4b–c illustrates images corresponding to successive magnifications of a grain located in the region labeled as “VO_*x*_ (I)” showing characteristic lattice distances and angles of planes observable along the [120] VO_2_(M) zone axis. They are identified by the combined study of both HRTEM images and their respective fast Fourier transforms (FFTs), which are spectra equivalent to the electron diffraction pattern of the area. Furthermore, EELS spectroscopy analyses carried out in different areas of sample T100_550_10 (Figure 4d,e) confirm the V^4+^ valence state (VO_2_) of this first region. It is characterized by EELS signatures with higher energy loss of the V-L_2,3_ white lines (the L3 position at 516.2 eV), the presence of two peaks in the O-K pre-edge region that correspond to the t_2g_ (around 528.1 eV) and e_g_ (around 530.7 eV) states, as well as the shoulders visible on the left sides of the L3 and L2 peaks (indicated by arrows).^52,53^ In contrast, the EELS signal recorded in region II (lower energy shift of the V-L_2,3_ lines, a single peak in the O-K pre-edge region) fits more with those reported for the V^2+^^53,54^ and V^3+^^52,54^ valence states, the latter being consistent with what was previously evidenced by GIXRD. This evidences the presence of the V_2_O_3_ phase in this sample at regions close to the substrate and therefore confirms the previous hypothesis that the oxidation process originates from the surface toward the interior of the film.

On the other hand, sample T100_550_30 shows considerably different characteristics than the previous one. Although two regions can also be distinguished, they are not so obvious in this case, since both their morphology and thickness do not follow an established pattern (see Figure 5a). Successive magnifications of a boundary zone between these two regions show interplanar spacings and angles that match with the [124] VO_2_(M) orientation (Figure 5b,c). However, differently from sample T100_550_10, it appears that the region denoted as VO_*x*_ (II) is the one with the higher valence state oxide. Figure 5d,e shows the integrated EELS spectra collected in different regions of sample T100_550_30. They reveal signals corresponding to the valence state V^4+^ (VO_2_) and V^5+^ (V_2_O_5_) for regions furthest (spectra #1–3) and closest (spectrum #4) to the substrate, respectively (according to the trends evidenced in the literature). It becomes evident that the VO_2_(M) + V_2_O_5_ system evolves in a totally different way from the previous one, giving rise to more irregular and inhomogeneous coatings with grain sizes comparable to the total film thickness. This latter could somehow explain the presence of vanadium pentoxide close to the interface with the silicon substrate. In any case, it should not be forgotten that this sample is the only one that moves away from the trends observed for the rest of the annealed samples in terms of composition and the surface microstructure.

**Figure 6:**
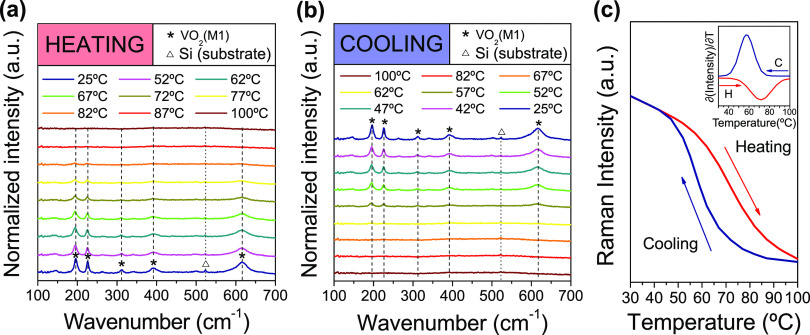
Raman spectra recorded for sample T200_550_45 at multiple temperatures between 25 and 100 °C during consecutive (a) heating and (b) cooling cycles. (c) Thermal evolution of the intensity of the VO_2_(M) Raman band at 194 cm^–1^ during heating (red) and cooling (blue) cycles. The inset shows the derivative of each kinetic thermochromic cycle (the derivative of the cooling is plotted in absolute values).

To explore the features of the structural phase transition (SPT) of the pure VO_2_/Si coating (sample T200_550_45, according to GIXRD studies), further Raman spectroscopy analyses were carried out at different temperatures between 25 and 100 °C for consecutive heating/cooling cycles (Figure 6). Figure 6a shows the progressive disappearance of the Raman bands associated with the monoclinic VO_2_ phase once *T*_c_ is exceeded during heating cycles. This gives way to the rutile VO_2_(R) phase, which is characterized by the absence of Raman signal (metallic behavior).^55^ The contrary trend is observed in Figure 6b, with a VO_2_(R) → VO_2_(M) phase change at lower critical temperatures during cooling. Figure 6c displays the MIT hysteresis resulting from the variation of the normalized Raman signal of the VO_2_(M) band at 194 cm^–1^ for consecutive heating/cooling cycles, highlighting a clear thermochromic response of the synthesized VO_2_/Si coating associated with the reversible monoclinic to rutile phase change. *T*_c_ values, estimated from the derivatives curves of the Raman intensity vs temperature plots with a Gaussian fit, show slightly higher temperatures than the conventional value reported for pure VO_2_ films during the heating cycle (∼68 °C). However, this shift toward higher temperatures was also observed in our previous work.^43^ In addition, these former experiments also revealed a hysteresis loop width (*W*_H_, given by *T*_c(heating)_ – *T*_c(cooling)_) of about 15 °C. In any case, it is worth mentioning that these parameters will be reassessed/compared once the optical and electrical characterization of the synthesized VO_2_-based films (including sample T200_550_45) will be addressed.

### 3.2 Functional Metal-to-Insulator Responses

#### 3.2.1 Vis–NIR Reflectance at Variable Temperature

**Figure 7:**
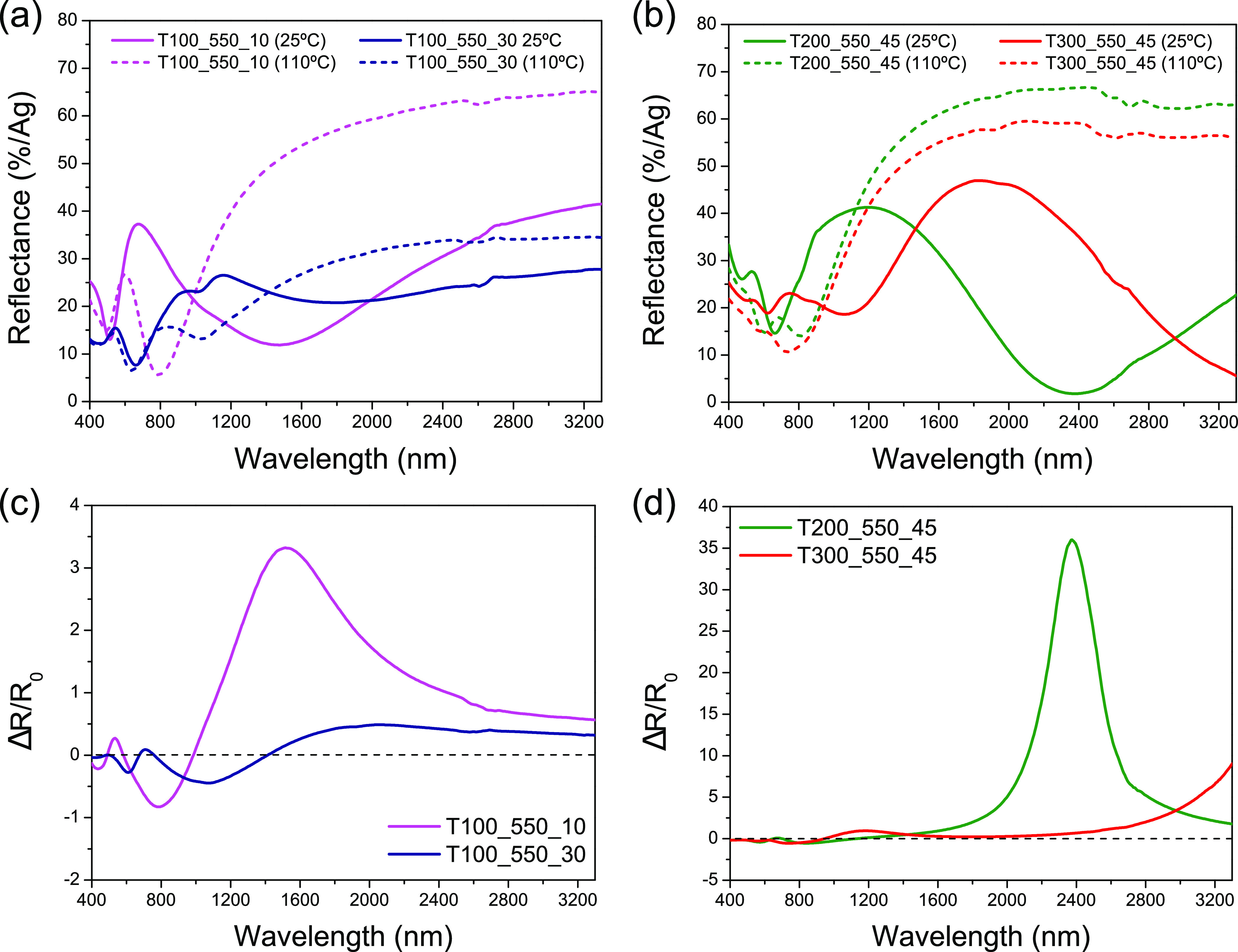
Reflectance spectra recorded at 25 °C (solid lines) and 110 °C (dashed lines) for samples (a) T100_550_10 and T100_550_30 and (b) T200_550_45 and T300_550_45. Rate of change in reflectance (*R* – *R*_0_) / *R*_0_ at 25 °C (*R*_0_) and 110 °C (*R*) for samples (c) T100_550_10 and T100_550_30 and (d) T200_550_45 and T300_550_45.

In the first instance, vis–NIR reflectance measurements (400–3300 nm) were conducted at 25 and 110 °C on samples annealed at 550 °C, covering each of the four possible scenarios: (i) VO_2_ + V_2_O_3_ mixtures (sample T100_550_10), (ii) VO_2_ + V_2_O_5_ mixtures (sample T100_550_30), (iii) pure VO_2_ (sample T200_550_45), and (iv) VO_2_ + V_6_O_13_ mixtures (sample T300_550_45). These spectra are shown in Figure 7a,b. Additionally, Figure 7c,d represents the rate of change in reflectance experienced by each of these samples defined by the Δ*R*/*R*_0_ ratio, where *R*_0_ is the reflectance values at 25 °C for the wavelength range previously established. The information extracted from all these measurements is summarized in Table 3, which lists the maximum change in reflectance (Δ*R*_max_), the wavelength at which this occurs (λ_max_), and the limiting wavelength beyond, which Δ*R* only takes positive values (λ_0_).

**Table 3:** Main Features of the Reflectance Changes Experienced by Different Annealed Samples in the Vis–NIR Range when Increasing Temperature^a^

sample	Δ*R*_max_ (%)	λ_max_ (nm)	λ_0_ (nm)
T100_550_10	40	1515	990
T100_550_30	11	2060	1415
T200_550_45	65	2375	1130
T300_550_45	50	3300	945

a Δ*R*_max_ indicates the maximum value taken by Δ*R* (which is given by the difference between the reflectance values at 110 and 25 °C, respectively), λ_max_ is the wavelength at which Δ*R*_max_ occurs, and λ_0_ denotes the limiting wavelength beyond which Δ*R* only takes positive values. The accuracies of reflectance (%) and wavelength (nm) values are ±0.5% and ±1 nm, respectively.

As generally discerned in Figure 7, the resulting optical responses for each of the examined samples are very diverse. On the one hand, sample T100_550_10 shows a maximum change in reflectance (Δ*R*_max_ = 40%) at 1515 nm, which is quite significant. By contrast, sample T100_550_30 presents substantially smaller reflectance variations over the whole wavelength range explored, reaching its maximum (Δ*R*_max_ = 11%) at further wavelengths (λ_max_ = 2060 nm). On another note, while the sample consisting exclusively of vanadium dioxide (i.e., T200_550_45) exhibits reflectance changes of more than 30% above 1600 nm (Δ*R*_max_ = 65% at 2375 nm), it is thought that the maximum Δ*R* for sample T300_550_45 would be rather centered within the mid-wavelength infrared (MWIR) spectral band (note that a maximum reflectance variation of 50% was registered at 3300 nm, which is the limiting wavelength for the spectral window considered). In any case, although, unlike what was observed for sample T100_550_30, most of the coatings show similar metallic responses at 110 °C, it should be stressed that sample T200_550_45 exhibits a higher NIR reflectance than the rest despite being thinner than T300_550_45. This could be attributed to the fact that it only consists of VO_2_. Likewise, the main difference between the optical responses of the latter two samples lies in their spectra at room temperature, which are relatively comparable in terms of their signature signals, but with a shift toward longer wavelengths in the case of T300_550_45.

Taking as a model the optical response associated with the VO_2_/Si coating (i.e., sample T200_550_45), it is possible to observe how the VO_*x*_ mixtures previously identified exhibit optical responses that, in one way or another, deviate from this ideality. For this purpose, special attention will be paid to the values taken by the parameters listed in Table 3. In this regard, the values of λ_max_ mark the onset of free-carrier dominated reflectance (free-carrier absorption typical of semiconductor systems) for measurements at 25 °C, while λ_0_ determines the wavelength from which effective reflectance modulation is achieved. On the one hand, sample T100_550_10 (VO_2_ + V_2_O_3_) exhibits Δ*R*_max_ at the lowest wavelength, resulting in reflectance modulations of about 20–40% between 1200 and 3300 nm. On the contrary, sample T100_550_30 formed by VO_2_ + V_2_O_5_ shows the weakest metallic response (*R* < 35% in the NIR) at 110 °C. This phenomenon, which can be directly attributed to the presence of pentoxide or to the formation of reduced amounts of dioxide, is considerably detrimental to the thermochromic properties of the coating.

Conversely, sample T300_550_45 (VO_2_ + V_6_O_13_) presents λ_0_ and λ_max_ values lower and higher, respectively, than those of sample T200_550_45. This somehow implies a larger application window, although at the cost of a decrease (although not as severe as that recorded for sample T100_550_30) of the NIR reflectance for the VO_2_(R) metallic phase. Similarly, this sample shows Δ*R* > 30% at wavelengths above 2500 nm, although at the expense of discrete changes in reflectance (10–20%) between 1000 and 2000 nm. In any case, it should be highlighted that the changes in NIR reflectance accomplished in this study, especially those of sample T200_550_45, are outstanding compared to the best values reported so far in the literature for similar VO_2_ coatings.^56−60^ Hence, it seems clear that both VO_2_ + V_2_O_3_ or VO_2_ + V_6_O_13_ mixtures could be advantageous for optical applications within specific infrared windows such as SWIR and MWIR, respectively. Also noteworthy is the fact that similar trends and optical responses were observed for the equivalent VO_*x*_ mixtures achieved at 475 °C (for more information, refer to the Supporting Information, Section II), again demonstrating the suitability of the selected thermal treatment window in terms of temperature and reaction times.

**Figure 8:**
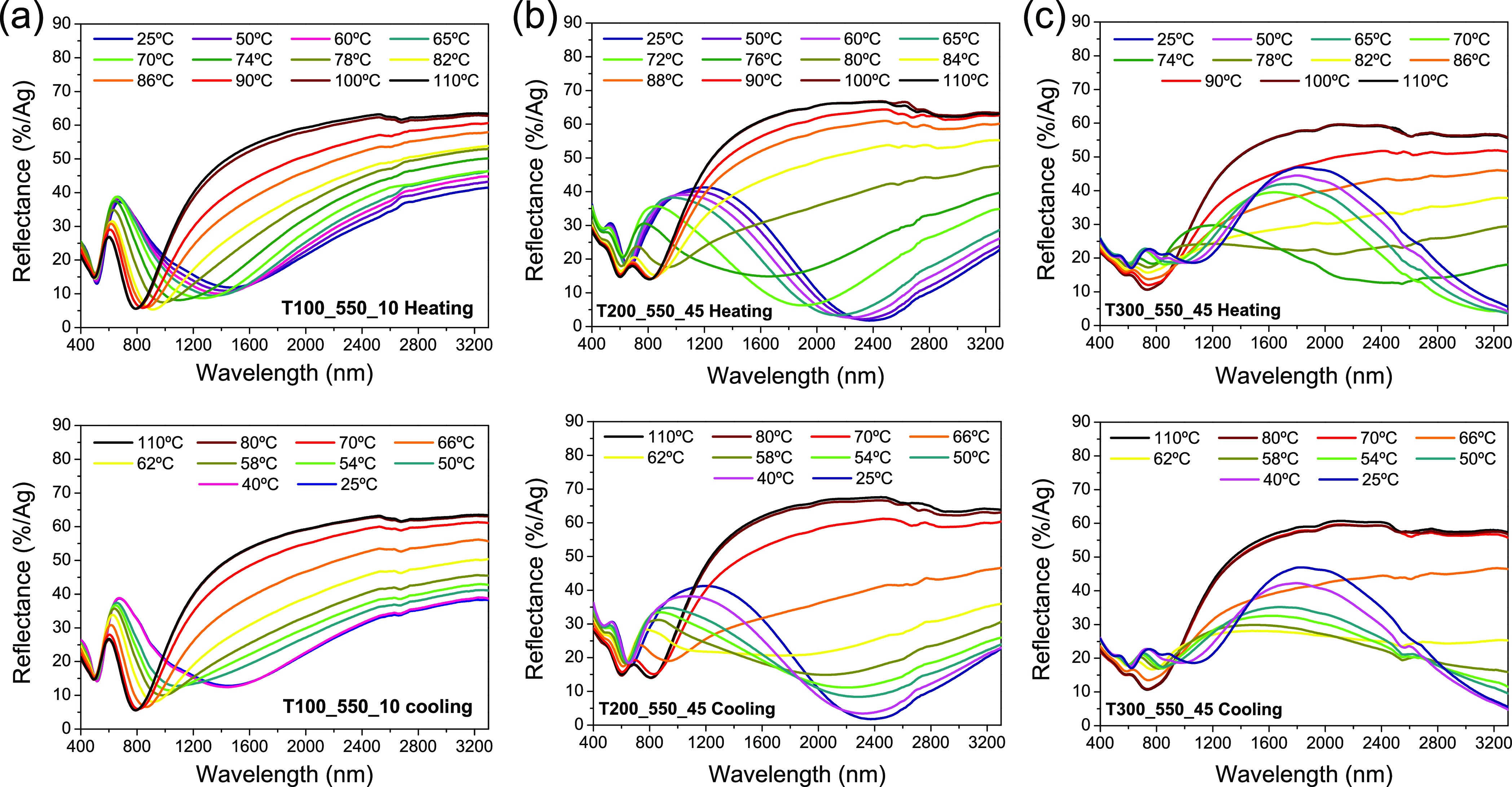
Vis–NIR reflectance spectra of samples (a) T100_550_10, (b) T200_550_45, and (c) T300_550_45, recorded gradually increasing (top) or decreasing (bottom) the temperature.

Figure 8 shows the reflectance changes experienced by the best 100, 200, and 300 nm thick samples achieved at 550 °C with 10 and 45 s during consecutive heating/cooling cycles at temperatures between 25 and 110 °C. Note that this outline allows a better appreciation of the progressive shift of absorption edge toward higher wavelengths for the semiconducting behavior linked to VO_2_(M) as both the film thickness and overall O/V ratio of the film increase. According to the literature, this phenomenon is rather due to the Burstein–Moss effect, which becomes more significant with decreasing the grain size as well as the appearance of impurities and defects.^61,62^ Likewise, it can be observed the great reflectance variations that these samples experience in the 400–3300 nm range at temperatures below and above *T*_c_ during heating and cooling cycles. Therefore, it seems clear that, for a better determination of the hysteresis loops, the thermal evolution of the reflectance for these samples should be performed at a fixed wavelength from λ_max_ onward.

**Figure 9:**
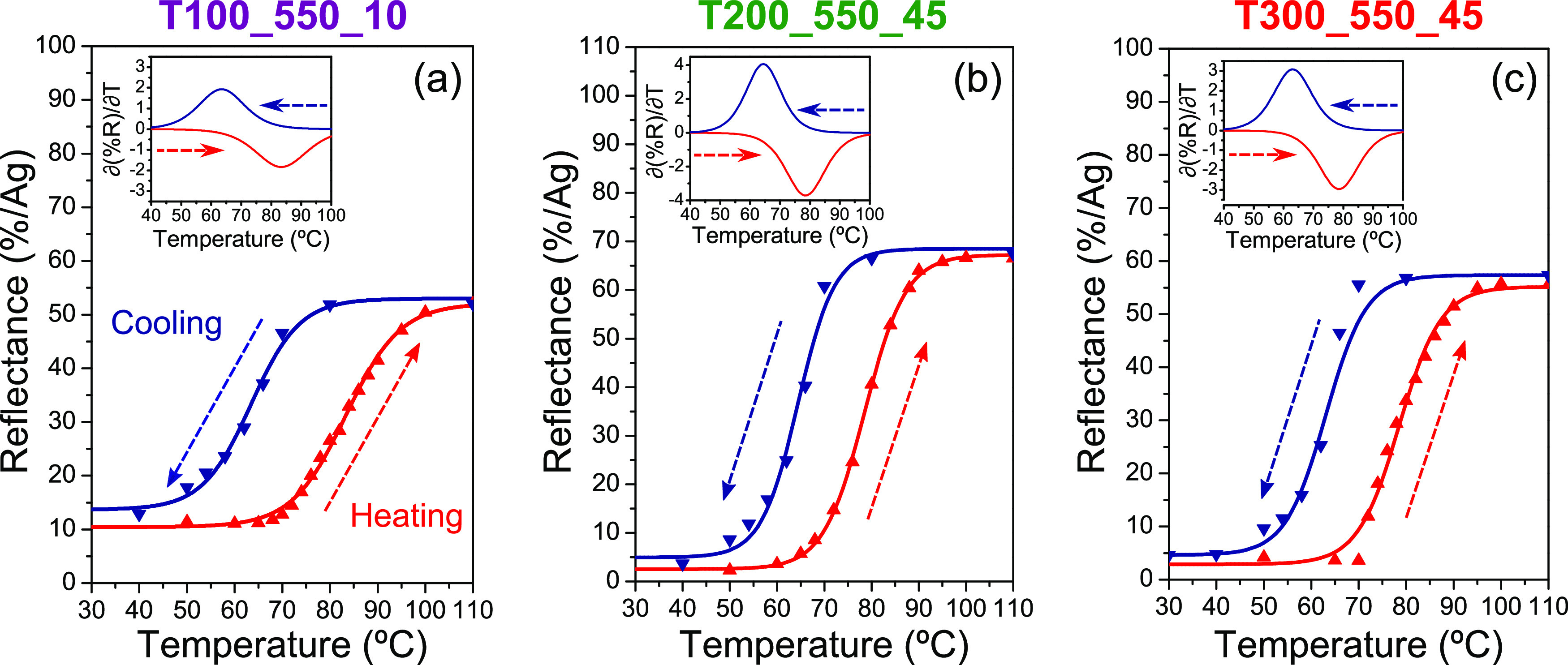
Thermal evolution of the optical reflectance of samples (a) T100_550_10, (b) T200_550_45, and (c) T300_550_45 evaluated at their λ_max_ during heating (red symbols) and cooling (blue symbols) cycles. Solid lines denote the sigmoidal fits (Boltzmann function) of the experimental results for each single kinetic cycle. The insets show the derivatives of such fits (the derivatives of the heating cycles are plotted in absolute values).

Figure 9 displays the kinetic evolution of the reflectance at λ_max_ for samples T100_550_10 (Figure 9a), T200_550_45 (Figure 9b), and T300_550_45 (Figure 9c) during consecutive heating and cooling cycles. Table 4 lists the transition temperatures, at heating (*T*_c(heating)_) and cooling (*T*_c(cooling)_), calculated from the derivatives of the sigmoidal fits (Boltzmann function) of the reflectance vs temperature curves, considering the peaks as the temperature of the minimum variation rate (insets in Figure 9), together with the width of the hysteresis loop (*W*_H_). In the first instance, it can be seen that moving away from the ideality of the VO_2_/Si system leads to an increase (which are more substantial for the specific case of VO_2_ + V_2_O_3_ mixtures) of *T*_c(heating)_ above 78 °C recorded for the sample T200_550_45, which is already high in itself. In contrast, *T*_c(cooling)_ values for this group of samples are almost similar (63–64 °C), resulting in wider hysteresis for mixtures of vanadium oxides. Generally, the increase in *W*_H_ is related to the presence of dopants, defects, or impurities,^63,64^ as well as the existence of additional energy barriers due to the residual stress associated with the rapid annealing treatments addressed here, which can delay the phase transition upon heating or cooling.^65^ Nevertheless, it should be noted that the hysteresis width for all these samples are consistent with those reported for VO_2_ polycrystalline films (*W*_H_ > 10 °C).^66^

**Table 4:** Main Features of the Thermochromic Hysteresis Loops Illustrated in Figure 9^a^

sample	*T*_c_ (heating) (°C)	*T*_c_ (cooling) (°C)	*W*_H_ (°C)
T100_550_10	83	63	20
T200_550_45	78	64	14
T300_550_45	79	63	16

a *T*_c_ (heating) denotes the temperatures of the MIT on heating, *T*_c_ (cooling) indicates the temperatures of the MIT on cooling, and *W*_H_ is the hysteresis loop width given by *T*_c_ (heating) – *T*_c_ (cooling). The accuracy of temperature values is ±0.5 °C.

Last but not least, it is worth highlighting once again the remarkable optical performances of samples T200_550_45 and T300_550_45, which not only exhibit changes in reflectance above 50% adapted for different wavelength ranges within the NIR window but also reflectance values close to 0% at room temperature (see Figure 9b,c). This makes such systems of potential interest for multiple optical applications (switches, filters, modulators, etc.).

#### 3.2.2 DC Electrical Resistivity vs Temperature

**Figure 10:**
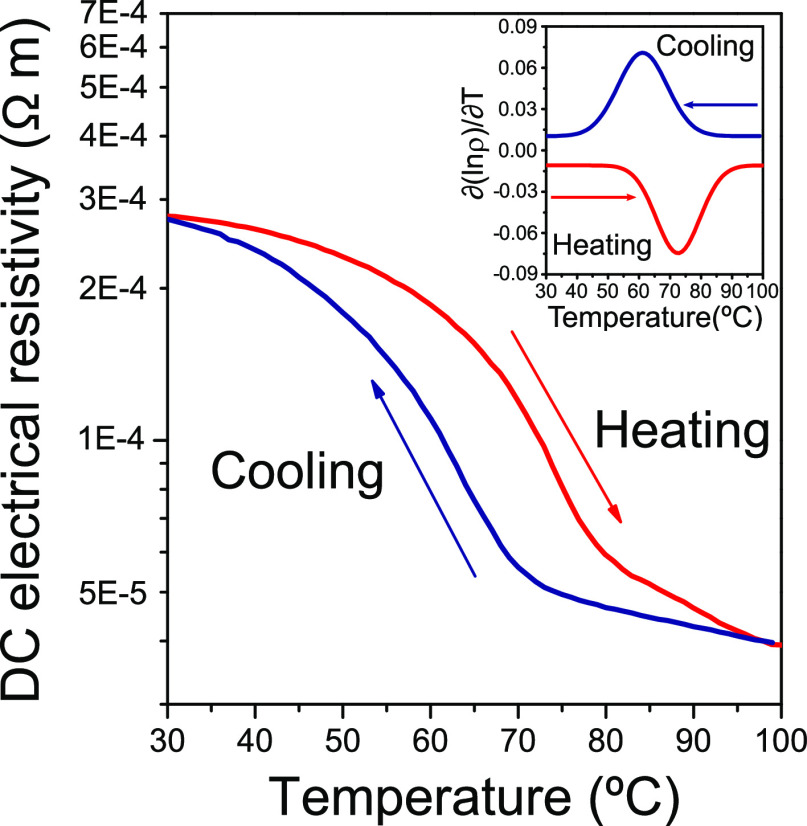
DC electrical resistivity vs temperature measured for sample T200_550_45 during heating (red) and cooling (blue) cycles. The inset shows the Gaussian fit for the derivative of each kinetic thermochromic cycle (the derivative of the cooling cycle is plotted in absolute values).

The comprehensive characterization of the fabricated coatings is culminated by performing resistivity vs temperature measurements to assess the features of their semiconductor-to-metal electrical transitions. In this sense, it should be mentioned that certain difficulties were encountered when carrying out such measurements given the substrate type (Si doped with P) as well as the electrical characteristics of some of the oxides formed in the VO_*x*_ mixtures (note that V_2_O_3_ exhibits room-temperature electrical conductivities almost six orders of magnitude higher than those of VO_2_^67^). This is why the 100 nm samples could not be measured. On the other hand, V_6_O_13_ also presents a conductivity at 25 °C three orders of magnitude higher than that of VO_2_.^68^ That is why, although the MIT hysteresis can be recorded for VO_2_ + V_6_O_13_ mixtures, the orders of magnitude of such drops in resistivity are practically negligible (for more details on the results obtained for these samples, refer to the Supporting Information, Section III). Thus, these findings were considered as not representative of the thermochromic performance associated with such coatings. Better results were obtained for the T200_550_45 sample (formed exclusively by VO_2_) as can be seen in Figure 10. However, given the above reasons, the registered resistivity drop was once more rather discrete (only ∼0.7 compared to 3–4 orders of magnitude expected for the resistivity drop in VO_2_ films according to the literature^69−71^). In any case, such measurements allowed the evaluation of the electrical MIT hysteresis of this sample.

### 3.3 Discussion on the Structure and Properties of MIT Variances for the VO_2_/Si System

At this point, the distinctive features of the different MIT hystereses (structural, optical, electrical) recorded for the same VO_2_/Si sample will be compared. Table 5 summarizes the characteristic parameters that evaluate the hysteresis loops of sample T200_550_45 extracted by RS, spectral reflectance, and resistivity measurements, all of them at variable temperature. In this sense, the noticeable difference between the features of each of these hystereses has been commonly associated with the different nature of the optical and structural MIT.^72^ Likewise, it has also been reported that the electrical properties in VO_2_ systems can be understood by analyzing the properties of the SPT.^73,74^ This explains why the structural and electrical characterizations of this sample evidence more similar results in terms of *T*_c_ and *W*_H_ values. In addition, the values of these parameters resulting from the resistivity vs temperature measurements are comparable to those reported in the literature.^72^ That is not the case for the *T*_c_ values obtained by reflectance measurements, which are abnormally high (even in contrast to what was evidenced in our most recent studies dealing with the fabrication of VO_2_-based coatings on glass substrates^75,76^). This feature has generally been attributed to deposition conditions and postprocessing,^77^ so that the size of the synthesized grains as well as their homogeneity could affect this phenomenon. Likewise, residual strain along the *c*-axis^78^ and oxygen adsorption^79^ can also promote this increase in MIT temperature. In any case, it was previously observed that structural and/or compositional heterogeneity leads to higher *T*_c(heating)_ (see Table 4).

**Table 5:** Summary of the Structural, Optical, and Electrical Features of the MIT Hysteresis for Sample T200_550_45^a^

technique	*T*_c_ (heating) (°C)	*T*_c_ (cooling) (°C)	*W*_H_ (°C)
Raman spectroscopy	72	57	15
vis–NIR reflectance	78	64	14
DC electrical resistivity	73	61	12

a The accuracy of temperature values is ±0.5 °C.

In the light of the foregoing, it has been demonstrated that the rapid and controlled oxidation in air of V-GLAD films deposited on silicon substrates gives rise to VO_2_-based coatings at temperatures between 475 and 550 °C and reaction times below 120 s. Thanks to the modulation of the layer thickness and the heat treatment parameters, it was possible to synthesize thermochromic coatings of different compositions exhibiting very good optical performances (Δ*R* = 30–65%) adapted to specific wavelengths within the NIR window. This fact makes the synthesized systems of great interest for applications in optical switches or filters. Likewise, the synthesis of pure VO_2_ films on silicon was also attained. On the basis of the great SPT evidenced by Raman spectroscopy (structural and electrical responses are closely linked), it is thought that the order of magnitude of resistivity drop resulting from the VO_2_/Si system could be significantly improved by using undoped silicon. Under this circumstance, the synthesized systems would also be applicable in a multitude of optoelectronic and electronic smart devices. It is therefore concluded that the strategies described throughout this work open up a more environmentally friendly (thermal annealing in the absence of reactive gases, liquid solutions, or catalysts without special pressure requirements) and economically viable (low thermal budget associated with moderate temperatures and relatively short oxidation times) pathway for the fabrication of high-performance VO_2_-based films on silicon platforms.

## 4 Conclusions

An advantageous strategy for the fabrication of high-performance VO_2_-based thermochromic films on silicon for application in smart devices is reported. This procedure involves the GLAD deposition of metallic vanadium films with thicknesses between 100 and 300 nm and their subsequent fast oxidations in an air atmosphere at 475–550 °C for reaction times between 1 and 120 s. By modulating the V-GLAD layer width and thermal treatment parameters, high VO_2_(M) yields are achieved. Comprehensive characterizations of the structure and composition of the fabricated coatings by SEM and TEM electron microscopies, Raman spectroscopy, and X-ray diffraction show that all samples are, in most cases, composed of mixtures of VO_2_(M) and other vanadium oxides such as V_2_O_3_, V_6_O_13_, or V_2_O_5_. A 200 nm thick pure VO_2_/Si film is also synthesized at 550 °C for 45 s. On the other hand, structural, optical, and electrical analyses carried out at different temperatures between 25 and 110 °C allow to evaluate the thermochromic performance of such coatings. In this sense, vis–NIR reflectance measurements evidence changes in reflectance between insulator-metallic states sometimes higher than those reported so far for similar VO_2_/Si systems (note that several samples exhibit Δ*R* = 30–65% in the NIR range) with a remarkable maximum variation of 65% reached for the pure VO_2_ film at a wavelength of 2375 nm. By controlling the structure and composition of the coatings, effective tuning of the wavelength window at which the Δ*R*_max_ peaks in VO_2_ + V_2_O_3_ or V_6_O_13_ mixtures can also be achieved. The MIT hysteresis loops recorded by the optical characterization of these samples reveal *T*_c(heating)_ values up to 15 °C above the expected value for pure VO_2_ (∼68 °C), becoming higher for mixtures of vanadium oxides. This phenomenon is associated to several causes such as deposition and annealing processes, the presence of residual stress, defects, dopants, etc. Conversely, although the electrical characterization of most of VO_2_-based systems fabricated is hindered by several factors (use of doped silicon, formation of other high-conductivity vanadium oxides), the electrical MIT hysteresis for the VO_2_/Si system is unraveled, emphasizing lower *T*_c_ than those associated with the optical MIT, but similar to those registered through variable temperature Raman spectroscopy (structural MIT). These promising results not only demonstrate the feasibility of the alternative methodologies addressed but also the appeal of the manufactured systems for their integration into a multitude of optical, optoelectronic, and/or electronic smart devices.

## Supporting Information Available

Section I (topographic SEM micrograph for sample T100_550_1), section II (vis–NIR reflectance spectra at 25 and 110 °C for samples annealed at 475 °C), and section III (electronic features of the MIT for VO_2_ + V_6_O_13_ mixtures) (PDF)
