# Supplementary material for: Facile Fabrication of High-Performance Thermochromic VO2-Based Films on Si for Application in Phase-Change Devices
Source: Chem Mater. 2023 May 30;35(11):4435–48. doi: 10.1021/acs.chemmater.3c00613 (PMC10268973; doi:10.1021/acs.chemmater.3c00613)
Supplement: Supplementary file 1 — cm3c00613_si_001.pdf [file cm3c00613_si_001.pdf]

Facile fabrication of high-performance thermochromic VO_2-based films on Si for application in phase-change devices

Antonio J. Santos[1,2,3,], Nicolas Martin[3], Juan J. Jiménez[1,2], Rodrigo Alcántara[1,4], Samuel Margueron[3], Andrea Casas-Acuña[1,2], Rafael García[1,2], Francisco M. Morales[1,2]*

[1] IMEYMAT: Institute of Research on Electron Microscopy and Materials of the University of Cádiz, E-11510, Puerto Real, Spain.

[2] Department of Materials Science and Metallurgic Engineering, and Inorganic Chemistry, Faculty of Sciences, University of Cádiz, E-11510 Puerto Real, Spain.

[3] Université de Franche-Comté, CNRS, Institut FEMTO-ST, F-25000 Besançon, France.

[4] Department of Physical Chemistry, Faculty of Sciences, University of Cádiz, E-11510 Puerto Real, Spain.

[*] Corresponding author: antonio.santos@uca.es

ABSTRACT: This work reports on an alternative and advantageous procedure to attain VO_2-based thermochromic coatings on silicon substrates. It involves the sputtering of vanadium thin films at glancing angles and their subsequent fast annealing in air atmosphere. By adjusting

thickness and porosity of films as well as the thermal treatment parameters, high VO_2(M) yields were achieved for 100, 200 and 300 nm thick layers treated at 475 and 550°C for reaction times below 120 s. Comprehensive structural and compositional characterization by Raman spectroscopy, X-ray diffraction and scanning-transmission electron microscopies combined with analytical techniques such as electron energy-loss spectroscopy bring to the fore the successful synthesis of VO_2(M) + V_2O_3/V_6O_{13}/V_2O_5 mixtures. Likewise, a 200 nm thick coating consisting exclusively of VO_2(M) is also achieved. Conversely, the functional characterization of these samples is addressed by variable temperature spectral reflectance and resistivity measurements. The best results are obtained for the VO_2/Si sample with changes in reflectance of 30–65% in the near-infrared at temperatures between 25ºC and 110ºC. Similarly, it is also proven that the achieved mixtures of vanadium oxides can be advantageous for certain optical applications in specific infrared windows. Finally, the features of the different structural, optical and electrical hysteresis loops associated with the metal-insulator transition of the VO_2/Si sample are disclosed and compared. These remarkable thermochromic performances hereby accomplished highlight the suitability of these VO_2-based coatings for applications in a wide range of optical, optoelectronic and/or electronic smart devices.

Supporting Information

SECTION I: Topographic SEM micrograph for sample T100_550_1

This section includes the topographic SEM micrograph for sample T100_550_1 (**Fig. S1**), which, for reasons related to the organization and arrangement of the figures, was not included in the main manuscript. The average grain size for this sample is 35 ± 9 nm.

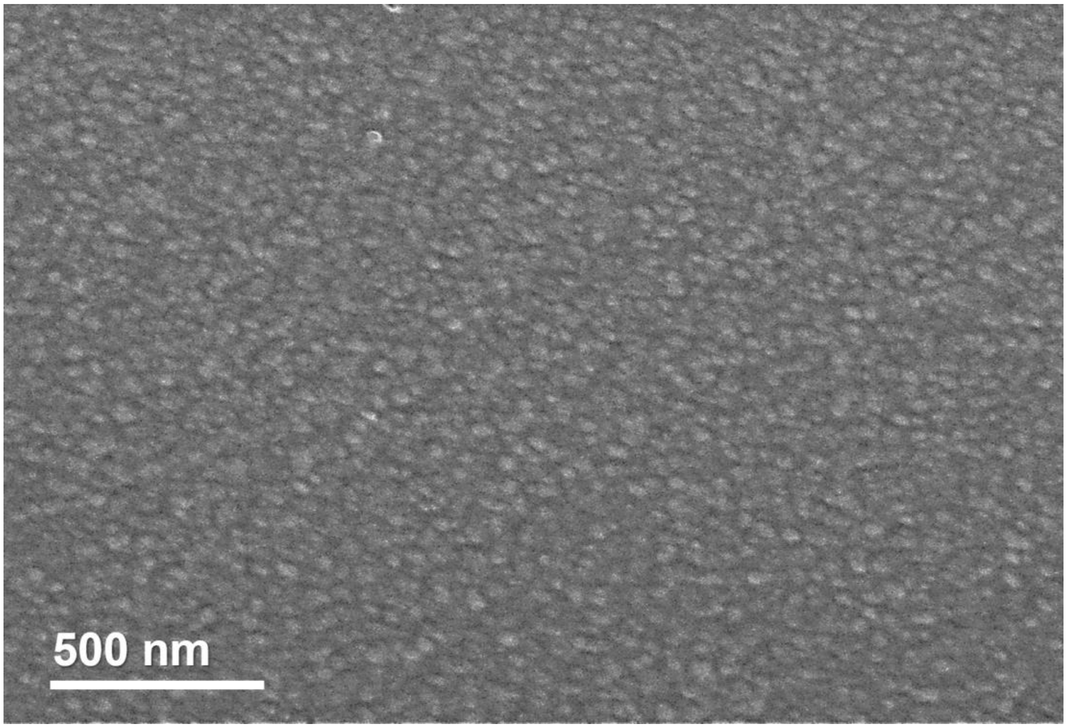

Fig. S1. Topographic SEM micrograph for sample T100_550_1.

SECTION II: Vis-NIR reflectance spectra at 25ºC and 110ºC for samples annealed at 475ºC

This section contains the reflectance spectra of the VO_2 + V_2O_3 (sample T100_475_60) and VO_2 + V_6O_{13} (samples T200_475_90 and T300_475_120) mixtures attained at 475ºC which, for reasons related to the organization and arrangement of the figures, were not included in the manuscript.

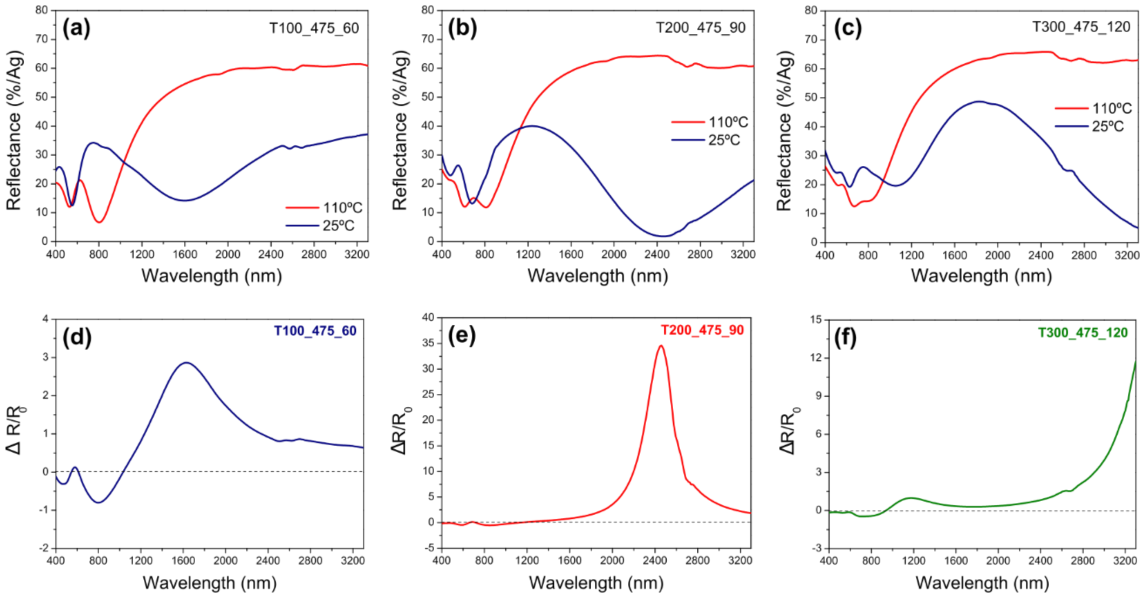

Fig. S2. Reflectance spectra recorded at 25ºC (blue) and 110ºC (red) for samples (a) T100_475_60, (b) T200_475_90 and (c) T300_475_120. Rate of change in reflectance $(R - R_0)/R_0$ at 25ºC (R_0) and 110ºC (R) for samples (d) T100_475_60, (e) T200_475_90 and (f) T300_475_120.

Table S1. Main features of the reflectance changes experienced by different annealed samples in the vis-NIR range when increasing temperature. ΔR_{max} indicates the maximum value taken by ΔR (which is given by the difference between the reflectance values at 110ºC and 25ºC, respectively); λ_{max} is the wavelength at which ΔR_{max} occurs; and λ_0 denotes the limiting wavelength beyond which ΔR only takes positive values. The accuracies of reflectance (%) and wavelength (nm) values are ±0.5% and ±1 nm, respectively.

**Sample**	**ΔR_{max} (%)**	**λ_{max} (nm)**	**λ_0 (nm)**
T100_475_60	41	1630	1035
T200_475_90	63	2460	1130
T300_475_120	58	3300	940

SECTION III: Electronic features of the MIT for VO_2 + V_6O_{13} mixtures

Fig. S3 shows the hysteresis loops for samples T200_475_90, T300_475_120 and T300_550_45 through resistivity *vs.* temperature measurements. Note that, despite the limited resistivity drops recorded, it was possible to evaluate the features of the electrical MIT for VO_2 + V_6O_{13} mixtures. **Table S2** lists the transition temperatures (T_c) during heating and cooling cycles, which were calculated from the derivative curves of the resistivity *vs*. temperature plots (**Fig. S2**) by fitting them with a Gaussian function and considering the peaks as the temperature of the minima variation rates, along with the hysteresis loop width (W_H) given by T_c (heating) – T_c (cooling).

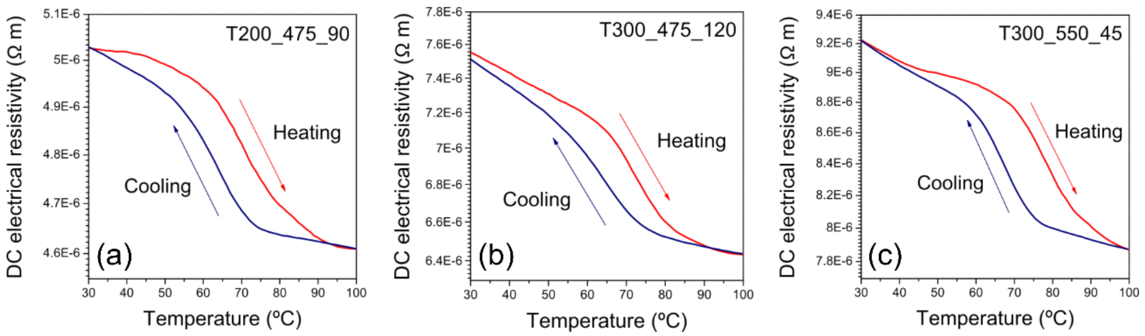

Fig. S3. DC electrical resistivity *vs.* temperature measured for samples (a) T200_475_90, (b) T300_475_120 and (c) T300_550_45 during heating (red) and cooling (blue) cycles.

Table S2. Main electronic features of the MIT extracted from the resistivity *vs*. temperature measurements carried out on VO_2 + V_6O_{13} mixtures. The accuracy of temperature values is ±0.5ºC.

Sample	T_c (ºC) heating	T_c (ºC) cooling	W_H (ºC)
T200_475_90	71	64	7
T300_475_120	72	64	8
T300_550_45	78	67	13
